# Factors Influencing Symptom Severity at Discharge after Lobectomy and Sublobar Resection Through Video-assisted Thoracoscopic Surgery

**DOI:** 10.1093/icvts/ivaf170

**Published:** 2025-08-14

**Authors:** XiaoJuan Yang, Qian Zhang, Cuiling Ye, Yalan Cheng, Jianwei Wu, Yi Liang, Jianwei Su

**Affiliations:** Department of Cardiothoracic Surgery, Zhongshan City People’s Hospital, Zhongshan, Guangdong 528403, China; Department of Cardiothoracic Surgery, Zhongshan City People’s Hospital, Zhongshan, Guangdong 528403, China; Department of Cardiothoracic Surgery, Zhongshan City People’s Hospital, Zhongshan, Guangdong 528403, China; Department of Cardiothoracic Surgery, Zhongshan City People’s Hospital, Zhongshan, Guangdong 528403, China; Department of Cardiothoracic Surgery, Zhongshan City People’s Hospital, Zhongshan, Guangdong 528403, China; Department of Cardiothoracic Surgery, Zhongshan City People’s Hospital, Zhongshan, Guangdong 528403, China; Department of Cardiothoracic Surgery, Zhongshan City People’s Hospital, Zhongshan, Guangdong 528403, China

**Keywords:** video-assisted thoracoscopic surgery (VATS), patient-reported outcomes, symptom severity, postoperative management, personalized care

## Abstract

**Objectives:**

This study investigates factors affecting symptom severity at discharge in patients who have undergone lobectomy and sublobar resection via video-assisted thoracoscopic surgery for pulmonary nodules, including both benign and malignant cases.

**Methods:**

This retrospective analysis utilized data from a patient cohort in a randomized controlled trial at Zhongshan City People’s Hospital. Symptom severity was assessed using the validated Perioperative Symptom Assessment for Lung Surgery questionnaire at 4 time points. Patients were grouped by discharge-day symptoms: Alert (scores >3) and No Alert. Symptom scores were further compared between pathology subgroups: lung cancer and benign/others. Mann-Whitney tests and repeated measures analysis of variance were used to compare symptom trajectories between groups. Univariate and multivariate logistic regression analyses were employed to identify factors associated with symptom severity at discharge.

**Results:**

Two hundred and forty-three patients were included in the analysis. The Alert group showed slower postoperative symptom improvement compared to the No Alert group (*P* < .05). Logistic regression analysis identified several key factors associated with symptom severity at discharge, including age, gender, smoking history, FEV1% (Forced Expiratory Volume in 1 second as a percentage of the predicted value), right upper lobe involvement, tumour stage, in-hospital complications, and length of stay after operation. In-hospital complications were significantly associated with increased severity of symptoms at discharge, including disturbed sleep, fatigue, drowsiness, and sadness.

**Conclusions:**

Multiple patient-specific and surgical factors influence postoperative symptom severity at discharge. These findings identify key factors associated with symptom severity and may inform future personalized management strategies following lung surgery.

**Clinical Registration Number:**

ClinicalTrials.gov; NCT05990946; https://clinicaltrials.gov/study/NCT05990946.

## Graphical Abstract

**Figure:**
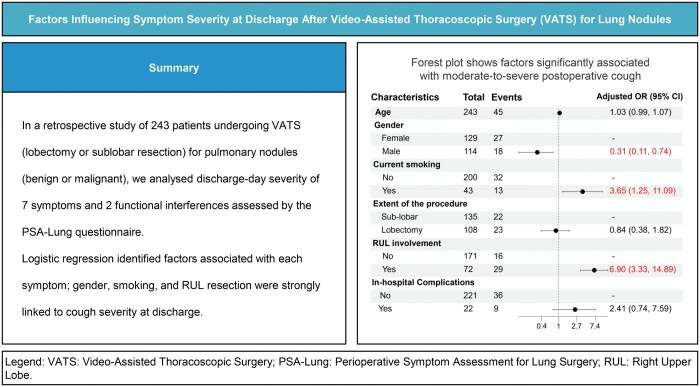

## INTRODUCTION

Lung cancer remains one of the most common and deadliest cancers worldwide, and is the leading cause of cancer-related mortality in China.^1^ Video-assisted thoracoscopic surgery (VATS), a less invasive alternative to thoracotomy, has shown advantages such as reduced pain and shorter recovery.^2–7^ However, many patients still experience postoperative symptoms like pain, fatigue, dyspnoea, and disturbed sleep, which can hinder recovery and affect quality of life.^8–11^

Previous studies have examined symptom burden after VATS, but most focused on isolated factors.^8^^,^^10^ Comprehensive evaluations of symptom severity and its associated factors at discharge remain limited.^12^^,^^13^

This study aims to address these gaps by analysing preoperative and postoperative symptom scores and patient characteristics to identify factors influencing symptom severity at discharge after VATS. These findings aim to identify patient- and surgery-related factors associated with postoperative symptom severity, and may inform future research on personalized symptom management strategies.

## METHODS

### Participants

This study is a retrospective analysis using data extracted from the patient cohort of a randomized controlled trial (NCT05990946) conducted at Zhongshan City People’s Hospital from December 2023 to May 2024. The trial included patients diagnosed with pulmonary nodules who underwent VATS. The inclusion criteria were: (1) aged between 18 and 80 years; (2) clinically diagnosed with pulmonary nodules and underwent VATS (lobectomy or sublobar resection, including wedge resection and segmentectomy); (3) completed the Perioperative Symptom Assessment for Lung Surgery (PSA-Lung) questionnaire, which demonstrates good sensitivity and validity in assessing postoperative symptoms after thoracic surgery.^14^ The PSA-Lung questionnaire assesses 7 symptoms (pain, cough, shortness of breath, disturbed sleep, fatigue, drowsiness, and sadness) and 2 functional interferences (walking ability and general activities), with scores ranging from 0 to 10, where higher scores indicate greater severity or interference. The questionnaire is provided in File S1. Exclusion criteria were: (1) severe comorbidities, such as cardiovascular disease or chronic obstructive pulmonary disease, that could impair postoperative recovery; (2) history of preoperative chemotherapy, multiple primary lung cancer for the second operation, history of other cancers, or cancer recurrence; (3) incomplete key data.

### Data collection

Symptom scores (via PSA-Lung) were recorded at 4 time points: preoperation (Pre-op), postoperative day 1 (POD1), discharge day, and 4 weeks postdischarge. Data were collected via app-based questionnaires with reminders. Clinical variables included demographics (age, gender, body mass index [BMI], education, smoking), comorbidities, FEV1%, surgical details (procedure type, resected lobe, lymphadenectomy, duration), tumour features (location, histology, stage), and postoperative length of stay. Tumour stage was treated as a categorical variable, with an additional category (“benign or others”) assigned to patients without malignant tumours to ensure complete model inclusion without bias. Complications were classified using the Clavien-Dindo classification system.^15^ Postdischarge pulmonary complications and unplanned readmissions were assessed within 30 days after hospital discharge. Data were securely stored in the Research Electronic Data Capture platform at Zhongshan City People’s Hospital.

### Ethics statement

This study was conducted in accordance with the Declaration of Helsinki (as revised in 2013) and was approved by the Ethics Committee of Zhongshan City People’s Hospital (Approval Number: K2022-285). Written informed consent was obtained from all participants. All data were anonymized, and strict confidentiality measures were implemented during data storage and analysis.

In accordance with the WMA Declaration of Taipei, the research data were securely stored in a dedicated database (Research Electronic Data Capture system) for potential future use. The establishment and continued use of this data repository were approved and monitored by the institutional ethics committee.

### Outcome measures

The primary outcome of this study was the identification of demographic and clinical factors associated with moderate-to-severe symptom severity at discharge, based on the PSA-Lung questionnaire. Symptom scores were classified as mild (0-3) or moderate-to-severe (4-10) according to prior studies.^10^^,^^16^

Secondary analyses included comparison of symptom trajectories across 4 perioperative time points (Pre-op, POD1, discharge, and 4 weeks postdischarge) between the Alert group (any discharge-day symptom score >3) and the No Alert group (all scores ≤3), and subgroup comparisons between patients with lung cancer and those with benign or other tumours.

### Data analysis

Data analysis was conducted using R software (version 4.2.2). Continuous variables were reported as mean ± SD, and categorical data as frequency (%). Mann-Whitney tests compared symptom severity between groups. Symptom trajectories were analysed using repeated measures analysis of variance (ANOVA).

Logistic regression identified predictors of discharge-day symptom severity. Variables were selected using backward stepwise regression based on Akaike information criterion minimization. Evaluated factors included age, gender, BMI, educational level, smoking status, comorbidities, FEV1%, extent of the procedure, right upper lobe (RUL) involvement, systematic dissection, operation time, tumour pathologic stage, in-hospital complications, and length of stay.

Categorical variables with low frequencies were recategorized: Smoking status was dichotomized, educational levels were merged into 2 categories, and lymphadenectomy type was combined into systematic dissection. Tumour pathologic stage was simplified into 3 categories: benign or others, stage I, and stages II-III. RUL involvement was defined as resection involving the RUL based on its clinical significance.^17^^,^^18^

## RESULTS

### Participant characteristics

**Figure 1.**
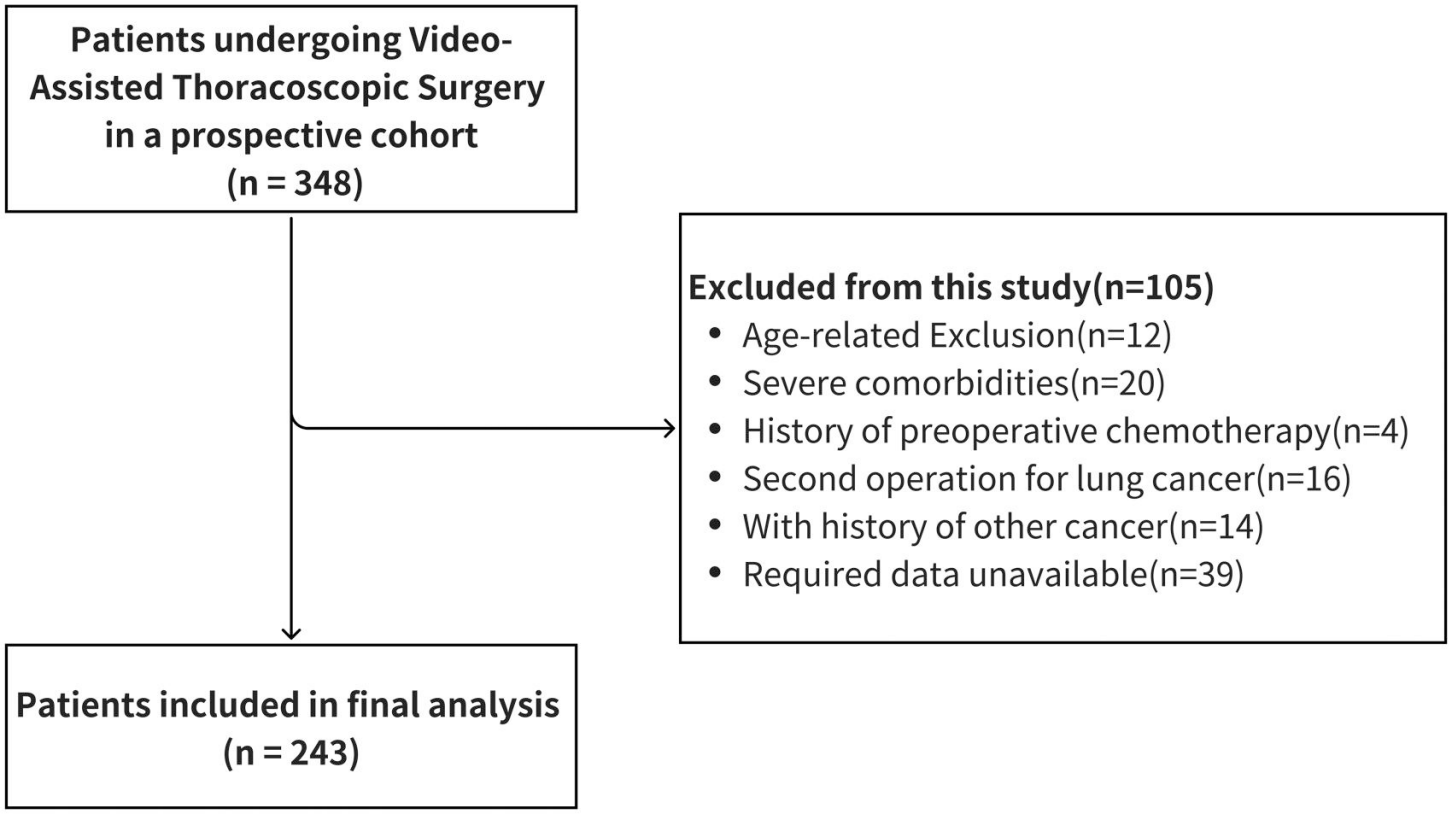
Patient Selection Flowchart

Of 348 screened patients, 243 were included; 105 were excluded due to age (*n* = 12), comorbidities (*n* = 20), prior chemotherapy (*n* = 4), repeat surgery (*n* = 16), other cancers (*n* = 14), or incomplete data (*n* = 39) (**Figure 1**).

The cohort had a mean age of 56.0 ± 10.6 years; 53.1% were female. Most were never-smokers (65.0%); 17.7% were current smokers. Mean BMI was 23.8 ± 3.6; mean FEV1% was 92.4 ± 16.4. Sublobar resections accounted for 55.6% of surgeries. Among the included patients, 203 were diagnosed with nonsmall cell lung cancer (NSCLC), including 192 adenocarcinomas and 11 squamous cell carcinomas. The remaining 40 had benign or nonprimary pulmonary lesions, such as inflammatory nodules (*n* = 21), hamartomas (*n* = 9), infectious granulomas (*n* = 3), fibrotic or scar nodules (*n* = 3), meningothelial-like nodules (*n* = 3), and a solitary pulmonary metastasis from an extrapulmonary malignancy (*n* = 1). Most nodules were stage I NSCLC (62.1%). Mean operation time was 137.5 ± 63.3 minutes; median hospital stay was 5.0 ± 3.9 days. In-hospital complications occurred in 9.1% of cases. Postdischarge pulmonary complications and unplanned readmissions within 30 days were observed in 2.5% and 4.1% of patients, respectively. A detailed summary of patient characteristics is provided in **Table 1**.

**Table 1.** Summary of Patient Characteristics

Mean ± SD or *n* (%)	*N* = 243
**Gender**	
Female	129 (53.1)
Male	114 (46.9)
**Age, years**	56.0 ± 10.6
**BMI, kg/m^2^**	23.8 ± 3.6
**Smoking status**	
Yes	43 (17.7)
Never	158 (65.0)
Quit (more than 6Mos)	15 (6.2)
Not provided	27 (11.1)
**Educational level**	
Above middle school	119 (49.0)
Middle school or below	118 (48.6)
Not provided	6 (2.5)
**Comorbidities**	
None	167 (68.7)
Yes	76 (31.3)
**FEV1%, %**	92.4 ± 16.4
Missing	6
**Extent of the procedure**	
Lobectomy	108 (44.4)
Sublobar	135 (55.6)
**Resected lobe**	
Right upper lobe	57 (23.5)
Right middle lobe	12 (4.9)
Right lower lobe	44 (18.1)
Left upper lobe	70 (28.8)
Left lower lobe	44 (18.1)
Right lobes	14 (5.8)
Left lobes	2 (0.8)
**Type of lymphadenectomy**	
Systematic dissection	192 (79.0)
Sampling	49 (20.2)
Not performed	2 (0.8)
**Operation time, min**	137.5 ± 63.3
**Tumour histologic type**	
Adenocarcinoma	192(79.0)
Squamous carcinoma	11(4.5)
Benign or others	40(16.5)
**Tumour pathologic stage**	
Tis	25 (10.3)
I	151 (62.1)
II	13 (5.3)
III	14 (5.8)
Benign or others	40 (16.5)
**Length of stay after operation**	5.0 ± 3.9
**In-hospital complications**	
No	221 (90.9)
Yes	22 (9.1)
**Out-of-hospital complications**	
No	237 (97.5)
Yes	6 (2.5)
**30-day readmission**	
No	233 (95.9)
Yes	10 (4.1)

Abbreviations: BMI, body mass index; FEV1%, forced expiratory volume in the first second as a percentage of the predicted value.

### Symptom-change trajectories

Patients with incomplete symptom data at Pre-op or discharge were excluded. Final analysis included complete data across all time points, except 3 missing at 4 weeks.

Symptom burden peaked on POD1, improved by discharge, and further decreased at 4 weeks. Cough and dyspnoea showed slower recovery.

The Alert group showed significantly higher symptom scores at all time points (*P* < .05), except for dyspnoea at 4 weeks (*P* = .107). Repeated measures ANOVA confirmed significant group-time interactions for all symptoms (*P* < .05). Detailed symptom score comparisons are provided in **Table 2**.

**Table 2.** Comparison of Symptom Scores across Different Time Points between Alert and No Alert Groups (Median ± SD)

Time point	Group	Pain	Cough	Shortness of breath	Disturbed sleep	Fatigue	Drowsiness	Sadness	Walking ability	General activity
Pre-Op	Alert (*n* = 151)	0.0 ± 0.7	0.0 ± 1.2	0.0 ± 0.9	1.0 ± 2.6	0.0 ± 1.8	0.0 ± 1.7	0.0 ± 1.8	0.0 ± 0.9	0.0 ± 1.0
	No Alert (*n* = 92)	0.0 ± 0.4	0.0 ± 0.8	0.0 ± 0.8	0.0 ± 1.6	0.0 ± 1.0	0.0 ± 0.9	0.0 ± 1.6	0.0 ± 0.2	0.0 ± 0.4
	Total (*n* = 243)	0.0 ± 0.6	0.0 ± 1.1	0.0 ± 0.9	0.0 ± 2.4	0.0 ± 1.6	0.0 ± 1.5	0.0 ± 1.7	0.0 ± 0.7	0.0 ± 0.9
	*P*-value (between groups)	.040	.525	.713	.001	.004	.005	.045	.017	.023
POD1	Alert (*n* = 151)	6.0 ± 1.8	3.0 ± 2.1	3.0 ± 1.9	5.0 ± 2.6	5.0 ± 2.5	4.0 ± 2.5	3.0 ± 2.4	5.0 ± 1.9	5.0 ± 2.0
	No Alert (*n* = 92)	5.0 ± 1.6	2.0 ± 1.8	2.0 ± 1.7	3.0 ± 2.5	3.0 ± 1.9	2.0 ± 1.8	2.0 ± 1.7	4.0 ± 1.5	4.0 ± 1.8
	Total (*n* = 243)	5.0 ± 1.8	3.0 ± 2.0	3.0 ± 1.9	5.0 ± 2.7	4.0 ± 2.4	3.0 ± 2.4	2.0 ± 2.2	5.0 ± 1.8	5.0 ± 2.0
	*P*-value (between groups)	<.001	.003	.001	<.001	<.001	<.001	.016	<.001	<.001
Discharge day	Alert (*n* = 151)	3.0 ± 1.4	3.0 ± 1.4	3.0 ± 1.5	3.0 ± 2.0	3.0 ± 1.9	3.0 ± 1.9	2.0 ± 1.8	3.0 ± 1.6	3.0 ± 1.6
	No Alert (*n* = 92)	2.0 ± 0.9	2.0 ± 0.9	2.0 ± 1.0	2.0 ± 1.2	1.0 ± 1.1	1.0 ± 1.0	1.0 ± 0.9	1.0 ± 1.2	1.0 ± 1.1
	Total (*n* = 243)	3.0 ± 1.4	2.0 ± 1.3	2.0 ± 1.4	3.0 ± 1.9	2.0 ± 1.8	2.0 ± 1.8	1.0 ± 1.6	2.0 ± 1.6	2.0 ± 1.5
	*P*-value (between groups)	<.001	<.001	<.001	<.001	<.001	<.001	<.001	<.001	<.001
4 Weeks postdischarge	Alert (*n* = 148)	1.0 ± 1.2	2.0 ± 1.2	2.0 ± 1.6	1.0 ± 1.5	1.0 ± 1.4	1.0 ± 1.3	1.0 ± 1.3	1.0 ± 1.2	1.0 ± 1.2
	No Alert (*n* = 92)	0.0 ± 0.7	2.0 ± 1.1	2.0 ± 1.3	1.0 ± 0.8	1.0 ± 0.7	0.0 ± 0.7	0.0 ± 0.9	0.0 ± 0.7	0.0 ± 0.8
	Total (*n* = 240)	1.0 ± 1.1	2.0 ± 1.2	2.0 ± 1.5	1.0 ± 1.3	1.0 ± 1.2	0.0 ± 1.1	0.0 ± 1.2	1.0 ± 1.1	1.0 ± 1.1
	*P*-value (between groups)	<.001	.005	.107	<.001	.001	<.001	<.001	<.001	<.001
	*P*-value (Group*Time point)	<.001	.008	.002	.045	<.001	<.001	.022	<.001	<.001

Abbreviations: Pre-op, preoperation; POD1, postoperative day 1.

No significant differences in symptom scores were found between lung caner and benign/other tumour groups at any assessment time points (**Table S1**).

### Factors affecting symptom severity at discharge

The association of these 14 variables with the symptom severity at discharge was analysed using both univariate and multivariate logistic regression models, with symptom severity classified as mild (0-3 points) or moderate-to-severe (4-10 points).

Pain: Male gender was associated with lower odds of severe pain compared to females (adjusted odds ratio [OR]: 0.55; 95% CI: 0.30-0.97; *P* = .041).

Cough: Male gender reduced the risk of severe cough (adjusted OR: 0.31; 95% CI: 0.11-0.74; *P* = .013), while current smoking (adjusted OR: 3.65; 95% CI: 1.25-11.09; *P* = .019) and RUL resection (adjusted OR: 6.90; 95% CI: 3.33-14.89; *P* < .001) significantly increased it.

Shortness of breath: Longer hospital stays were associated with greater severity (adjusted OR: 1.12; 95% CI: 1.03-1.21; *P* = .007).

Disturbed sleep: RUL resection (adjusted OR: 2.29; 95% CI, 1.23-4.28; *P* = .009) and tumour stages II-III (adjusted OR: 4.51; 95% CI, 1.42-14.35; *P* = .011) were significantly associated with disturbed sleep severity. In-hospital complications also increased the odds (adjusted OR: 3.24; 95% CI, 1.23-8.56; *P* = .017).

Fatigue: In-hospital complications were strongly associated with increased fatigue severity (adjusted OR: 4.80; 95% CI, 1.84-13.06; *P* = .002).

Drowsiness: In-hospital complications significantly increased the odds of drowsiness severity (adjusted OR: 6.13; 95% CI, 2.38-16.18; *P* < .001).

Sadness: Male gender had lower odds of higher sadness levels compared to females (adjusted OR: 0.39; 95% CI, 0.15-0.92; *P* = .039). In-hospital complications were strongly associated with increased sadness severity (adjusted OR: 6.86; 95% CI, 1.92-25.29; *P* = .003).

Walking ability and general activity: Walking interference was positively associated with age (adjusted OR: 1.07; 95% CI, 1.03-1.12; *P* = .002), educational level (adjusted OR: 3.06; 95% CI, 1.28-7.72; *P* = .014), FEV1% (adjusted OR: 0.98; 95% CI, 0.95-1.00; *P* = .047), and strongly with in-hospital complications (adjusted OR: 15.19; 95% CI, 5.21-49.18; *P* < .001). General activity interference was similarly strongly associated with age (adjusted OR: 1.04; 95% CI, 1.00-1.08; *P* = .035) and in-hospital complications (adjusted OR: 5.58; 95% CI, 1.65-19.75; *P* = .006).

Detailed results are presented in **Tables S2-S10**.

## DISCUSSION

Our study on patients undergoing VATS for pulmonary nodules revealed 3 key findings: (1) symptom severity peaked on POD1, followed by significant improvement by discharge and further recovery at 4 weeks postdischarge; (2) discharge-day symptom severity predicted recovery trajectories; and (3) multiple demographic and clinical factors influenced symptom severity at discharge.

Persistent symptoms after surgery significantly impact health-related quality of life and functional recovery.^19–23^ This highlights the importance of using validated tools like the PSA-Lung questionnaire for symptom evaluation.^24–26^ Our analysis identified multiple factors influencing symptom severity at discharge, several of which align with prior studies. Gender differences were observed, with males experiencing lower odds of severe pain, cough, and sadness, aligning with studies reporting gender-specific variations in recovery trajectories.^27–29^ Smoking history increased the risk of severe cough, reaffirming its detrimental role in respiratory recovery.^30^ RUL resection significantly predicted persistent cough, consistent with its known anatomical and functional impact.^17^^,^^18^ Additionally, advanced tumour stages were associated with more severe sleep disturbances, while prolonged hospital stays were linked to shortness of breath, likely due to limited rehabilitation during hospitalization. We expand on this knowledge by demonstrating that in-hospital complications play significant roles in influencing the severity of symptoms at discharge. Interestingly, factors such as lobectomy were significantly associated with severe fatigue at discharge in the univariate analysis, but this association did not persist in the multivariate analysis. This highlights the complexity of interactions between multiple factors, suggesting that various variables may contribute to recovery outcomes. These findings emphasize the importance of integrating demographic and clinical factors into personalized care plans and underscore the need for further research into the causal relationships between these factors and recovery outcomes. Additionally, subgroup analysis showed no differences in symptoms between NSCLC and benign/other tumour patients, suggesting similar postoperative recovery trajectories irrespective of pathology type.

These findings underscore the need for personalized care strategies focused on symptom management. Patients in the Alert group, particularly those with in-hospital complications or undergoing RUL resection, may benefit from closer monitoring and earlier interventions. For example, targeted respiratory rehabilitation programmes could be implemented to address persistent cough and dyspnoea, especially for patients with impaired pulmonary function.^31^ Smoking cessation should also be prioritized in preoperative care, as current smoking was strongly associated with severe postoperative cough. Gender differences in symptom severity were observed, with male patients showing lower odds of pain, cough, and sadness, highlighting the need for gender-specific approaches in symptom management and psychosocial support. Sadness in this study was defined as self-reported emotional low mood, which is a key aspect of psychological distress, a broader concept that encompasses symptoms like anxiety and depression. In some cultures, men are less likely to express emotional distress due to societal norms, which could explain why male patients in our study reported lower levels of severe sadness.^32^ Furthermore, addressing persistent symptoms like cough and shortness of breath is crucial, as these symptoms can significantly impede functional recovery and quality of life. Efficient risk stratification based on discharge-day symptoms and key predictors can help optimize care delivery, particularly in resource-limited healthcare settings.

This study presents several limitations: The relatively modest sample size and single-centre design may limit the generalizability of the findings. Multicentre studies involving diverse patient populations would provide more robust and externally valid data. The reliance on patient-reported outcomes for symptom assessment introduces the possibility of subjective bias, although the PSA-Lung questionnaire used in this study has demonstrated reliability and validity. Additionally, the retrospective design precluded the inclusion of data on socioeconomic status, social support, and psychological health, which are important factors that may influence symptom severity and recovery.^33^ All patients in this study underwent 2-port VATS and received patient-controlled analgesia, limiting the exploration of other perioperative variables that may affect recovery outcomes. In addition, sublobar resections were not further classified into segmentectomy versus wedge resection, which may have masked differential effects on postoperative symptoms due to varying anatomical extent and physiological impact. Furthermore, the inclusion of both benign and malignant pulmonary nodules may have introduced heterogeneity in symptom severity, potentially confounding the observed associations.

Future research should validate these findings in larger, multicentre cohorts and explore tailored interventions based on individual symptom profiles. Incorporating socioeconomic factors and leveraging artificial intelligence-driven predictive models could further enhance symptom management and long-term outcomes.

## CONCLUSION

In conclusion, this study provides valuable insights into the postoperative symptom trajectories of patients undergoing VATS for pulmonary nodules. By identifying key predictors of symptom severity and distinct patient groups, we lay the groundwork for more personalized and effective postoperative care strategies. Future research should focus on validating these findings in larger, diverse populations and developing targeted interventions to improve patient outcomes.

## Supplementary Material

ivaf170_Supplementary_Data

## Data Availability

The data underlying this article will be shared on reasonable request to the corresponding author.
