# Supplementary material for: Factors Influencing Symptom Severity at Discharge after Lobectomy and Sublobar Resection Through Video-assisted Thoracoscopic Surgery
Source: Interdiscip Cardiovasc Thorac Surg. 2025 Aug 14;40(8):ivaf170. doi: 10.1093/icvts/ivaf170 (PMC12368850; doi:10.1093/icvts/ivaf170)
Supplement: ivaf170_Supplementary_Data [file ivaf170_supplementary_data.zip › Supplementary file-PSA-lung-English version.pdf]

Perioperative Symptom Assessment - Lung Surgery (PSA-Lung)

How severe are your symptoms?

Patients may experience various symptoms and functional interference related to the disease or its treatment. We aim to assess the severity of the following symptoms you have experienced ***in the last 24 hours***. Please select a number from 0 (not present) to 10 (as bad as you can imagine) for each item to indicate the severity.

	**0 = Not present**										**10 = As bad as you can imagine**
	0	1	2	3	4	5	6	7	8	9	10
1. What is the severity of your **pain**?	○	○	○	○	○	○	○	○	○	○	○
2. What is the severity of your **cough**?	○	○	○	○	○	○	○	○	○	○	○
3. What is the severity of your **shortness of breath**?	○	○	○	○	○	○	○	○	○	○	○
4. What is the severity of your **disturbed sleep**?	○	○	○	○	○	○	○	○	○	○	○
5. What is the severity of your **fatigue (feeling tired/weary)**?	○	○	○	○	○	○	○	○	○	○	○
6. What is the severity of your **drowsiness**?	○	○	○	○	○	○	○	○	○	○	○
7. What is the severity of your **distress**?	○	○	○	○	○	○	○	○	○	○	○
8. What is the severity of your **walking difficulty**?	○	○	○	○	○	○	○	○	○	○	○
9. What is the severity of your **activity limitation**?	○	○	○	○	○	○	○	○	○	○	○

Copyright 2023 The Chongqing Medical University & Sichuan Cancer Hospital
All rights reserved.

A Brief Introduction of PSA-Lung

The Perioperative Symptom Assessment - Lung surgery (PSA-Lung) is a multisymptom patient-reported outcome (PRO) measure for clinical and research use. PSA-Lung is used to assess the severity of symptoms experienced by patients who have undergone lung surgery and their physical functional status as a result of these symptoms in daily living.

The PSA-Lung includes nine items (seven for symptoms and two for daily functioning). It asks patients to rate the most severe levels of symptoms and functional impairment on a 0-10 numeric rating scale:0 = not present and 10 = as bad as you can imagine. The recall period is set to 24 hours.

The PSA-Lung can be administered in both paper-and-pencil and web-based modes. The original language is Chinese, and the English version is also available. Patients can complete the PSA-Lung within 2 minutes, enabling a frequent assessment schedule. All items can be analyzed independently as continuous variables.

Please contact Dr. Qiuling Shi (qshi@cqmu.edu.cn) to order the PSA-Lung for use. PSA-Lung is free for projects with non-profit funding and non-funded academic research. Please discuss with the owners to use this in routine practice, commercial research, or reproduction in educational materials or other publications,.

References for validation

Relevant articles were submitted for publication and the preliminary results were announced at the 28th Annual Conference of the International Society for Quality of Life Research.

1. 28th Annual Conference of the International Society for Quality of Life Research. Qual Life Res. 2021;30 (Suppl 1):1–177. Title for "Measuring the Symptom Burden of Lung Surgery Patients: The Validity and Utility of the Perioperative Symptom Assessment for Lung Surgery (PSA-Lung)".

References for Application

1. Yang D, Hong Q, Zhao C, Mu J. Postoperative Patient-Reported Outcomes after Uniportal Video-Assisted Thoracoscopic Surgery Using the Perioperative Symptom Assessment for Lung Surgery Scale. Curr Oncol. 2022;29(10):7645-7654.
2. Yang D, Wei X, Hong Q, Zhao C, Mu J. Patient-Reported Outcome-Based Prediction for Postdischarge Complications after Lung Surgery. Thorac Cardiovasc Surg. 2023. Epub ahead of print.
