# Supplementary material for: Factors Influencing Symptom Severity at Discharge after Lobectomy and Sublobar Resection Through Video-assisted Thoracoscopic Surgery
Source: Interdiscip Cardiovasc Thorac Surg. 2025 Aug 14;40(8):ivaf170. doi: 10.1093/icvts/ivaf170 (PMC12368850; doi:10.1093/icvts/ivaf170)
Supplement: ivaf170_Supplementary_Data [file ivaf170_supplementary_data.zip › Supplementary Table S1 to S10.docx]

**Supplementary Table S1 to S10**

[Supplementary Table S1: Comparison of symptom scores across different time points between different pathology groups (Median ± SD)](#_Toc196137072)

[Supplementary Table S2: Univariate and Multivariate Analysis of Factors Influencing Symptom Severity at Discharge – Pain](#_Toc196137073)

[Supplementary Table S3: Univariate and Multivariate Analysis of Factors Influencing Symptom Severity at Discharge – Cough](#_Toc196137074)

[Supplementary Table S4: Univariate and Multivariate Analysis of Factors Influencing Symptom Severity at Discharge – Shortness of Breath](#_Toc196137075)

[Supplementary Table S5: Univariate and Multivariate Analysis of Factors Influencing Symptom Severity at Discharge – Disturbed Sleep](#_Toc196137076)

[Supplementary Table S6: Univariate and Multivariate Analysis of Factors Influencing Symptom Severity at Discharge – Fatigue](#_Toc196137077)

[Supplementary Table S7: Univariate and Multivariate Analysis of Factors Influencing Symptom Severity at Discharge – Drowsiness](#_Toc196137078)

[Supplementary Table S8: Univariate and Multivariate Analysis of Factors Influencing Symptom Severity at Discharge – Sadness](#_Toc196137079)

[Supplementary Table S9: Univariate and Multivariate Analysis of Factors Influencing Symptom Severity at Discharge – Walking Ability](#_Toc196137080)

[Supplementary Table S10: Univariate and Multivariate Analysis of Factors Influencing Symptom Severity at Discharge – General Activity](#_Toc196137081)

## **Supplementary Table S1: Comparison of symptom scores across different time points between different pathology groups** **(Median ± SD)**

| **Time Point** | **Group** | **Pain** | **Cough** | **Shortness of Breath** | **Disturbed Sleep** | **Fatigue** | **Drowsiness** | **Sadness** | **Walking Ability** | **General Activity** |
| --- | --- | --- | --- | --- | --- | --- | --- | --- | --- | --- |
| Pre-Op | Lung cancer(n=203) | 0.0 ± 0.6 | 0.0 ± 1.1 | 0.0 ± 0.9 | 0.0 ± 2.4 | 0.0 ± 1.5 | 0.0 ± 1.4 | 0.0 ± 1.7 | 0.0 ± 0.9 | 0.0 ± 0.7 |
|  | Benign or others (n=40) | 0.0 ± 0.3 | 0.0 ± 1.1 | 0.0 ± 0.7 | 0.0 ± 2.0 | 0.0 ± 2.0 | 0.0 ± 1.5 | 0.0 ± 1.7 | 0.0 ± 0.5 | 0.0 ± 0.7 |
|  | p-value (between groups) | 0.607 | 0.893 | 0.663 | 0.600 | 0.358 | 0.255 | 0.719 | 0.533 | 0.434 |
| POD1 | Lung cancer(n=203) | 5.0 ± 1.8 | 3.0 ± 2.0 | 3.0 ± 1.9 | 5.0 ± 2.7 | 4.0 ± 2.4 | 3.0 ± 2.3 | 2.0 ± 2.2 | 5.0 ± 2.0 | 5.0 ± 1.9 |
|  | Benign or others (n=40) | 5.0 ± 1.7 | 3.0 ± 2.0 | 3.0 ± 1.8 | 4.0 ± 2.7 | 4.0 ± 2.2 | 3.0 ± 2.4 | 2.0 ± 2.0 | 4.0 ± 1.7 | 4.0 ± 1.8 |
|  | p-value (between groups) | 0.653 | 0.600 | 0.872 | 0.412 | 0.238 | 0.753 | 0.969 | 0.171 | 0.052 |
| Discharge Day | Lung cancer(n=203) | 3.0 ± 1.5 | 2.0 ± 1.4 | 2.0 ± 1.4 | 3.0 ± 2.0 | 2.0 ± 1.9 | 2.0 ± 1.9 | 1.0 ± 1.7 | 2.0 ± 1.6 | 2.0 ± 1.6 |
|  | Benign or others (n=40) | 3.0 ± 1.1 | 3.0 ± 1.1 | 2.0 ± 1.2 | 2.5 ± 1.5 | 3.0 ± 1.5 | 2.0 ± 1.4 | 1.5 ± 1.2 | 2.0 ± 1.2 | 2.0 ± 1.1 |
|  | p-value (between groups) | 0.213 | 0.335 | 0.836 | 0.474 | 0.179 | 0.389 | 0.980 | 0.07 | 0.083 |
| 4 Weeks Post-Discharge | Lung cancer(n=200) | 1.0 ± 1.1 | 2.0 ± 1.2 | 2.0 ± 1.5 | 1.0 ± 1.4 | 1.0 ± 1.2 | 0.0 ± 1.2 | 0.0 ± 1.2 | 1.0 ± 1.1 | 1.0 ± 1.1 |
|  | Benign or others (n=40) | 1.0 ± 0.9 | 2.0 ± 1.1 | 2.0 ± 1.3 | 1.0 ± 0.9 | 1.0 ± 1.1 | 1.0 ± 1.0 | 0.5 ± 1.2 | 1.0 ± 1.0 | 1.0 ± 1.1 |
|  | p-value (between groups) | 0.832 | 0.187 | 0.557 | 0.162 | 0.060 | 0.236 | 0.659 | 0.057 | 0.072 |

SD: Standard Deviation; Pre-op: Post-operation; POD1: Postoperative Day

## **Supplementary Table S2: Univariate and Multivariate Analysis of Factors Influencing Symptom Severity at Discharge – Pain**

| **Characteristic** | **Univariable** | | | | | **Multivariable** | | | | |
| --- | --- | --- | --- | --- | --- | --- | --- | --- | --- | --- |
|  | **N** | **Event N** | **OR** | **95% CI** | **p-value** | **N** | **Event N** | **OR** | **95% CI** | **p-value** |
| **Age, years** | 243 | 75 | 1.01 | 0.98, 1.04 | 0.482 | 243 | 75 | 1.01 | 0.98, 1.04 | 0.438 |
| **Gender** |  |  |  |  |  |  |  |  |  |  |
| Female | 129 | 46 | — | — |  | 129 | 46 | — | — |  |
| Male | 114 | 29 | 0.62 | 0.35, 1.07 | 0.086 | 114 | 29 | 0.55 | 0.30, 0.97 | 0.041 |
| **BMI, kg/m^2^** | 243 | 75 | 1.02 | 0.95, 1.10 | 0.616 |  |  |  |  |  |
| **Educational level** |  |  |  |  |  |  |  |  |  |  |
| Middle school or below/Unknown | 124 | 37 | — | — |  |  |  |  |  |  |
| Above middle school | 119 | 38 | 1.10 | 0.64, 1.90 | 0.724 |  |  |  |  |  |
| **Current smoking** |  |  |  |  |  |  |  |  |  |  |
| No | 200 | 63 | — | — |  |  |  |  |  |  |
| Yes | 43 | 12 | 0.84 | 0.39, 1.71 | 0.644 |  |  |  |  |  |
| **Comorbidities** |  |  |  |  |  |  |  |  |  |  |
| No | 167 | 55 | — | — |  |  |  |  |  |  |
| Yes | 76 | 20 | 0.73 | 0.39, 1.32 | 0.301 |  |  |  |  |  |
| **FEV1%, %** | 243 | 75 | 1.01 | 0.99, 1.03 | 0.385 |  |  |  |  |  |
| **Extent of the procedure** |  |  |  |  |  |  |  |  |  |  |
| Sub-lobar | 135 | 44 | — | — |  | 135 | 44 | — | — |  |
| Lobectomy | 108 | 31 | 0.83 | 0.48, 1.44 | 0.515 | 108 | 31 | 0.55 | 0.28, 1.04 | 0.070 |
| **RUL involvement** |  |  |  |  |  |  |  |  |  |  |
| No | 171 | 49 | — | — |  |  |  |  |  |  |
| Yes | 72 | 26 | 1.41 | 0.78, 2.52 | 0.252 |  |  |  |  |  |
| **Systematic dissection** |  |  |  |  |  |  |  |  |  |  |
| No | 51 | 12 | — | — |  | 51 | 12 | — | — |  |
| Yes | 192 | 63 | 1.59 | 0.80, 3.36 | 0.205 | 192 | 63 | 1.71 | 0.82, 3.75 | 0.165 |
| **Operation time, minutes** | 243 | 75 | 1.00 | 1.00, 1.00 | 0.888 |  |  |  |  |  |
| **Tumor pathologic stage** |  |  |  |  |  |  |  |  |  |  |
| Benign or other tumor | 40 | 9 | — | — |  |  |  |  |  |  |
| Tis | 25 | 6 | 1.09 | 0.32, 3.51 | 0.889 |  |  |  |  |  |
| I | 151 | 52 | 1.81 | 0.83, 4.30 | 0.154 |  |  |  |  |  |
| II~III | 27 | 8 | 1.45 | 0.47, 4.44 | 0.512 |  |  |  |  |  |
| **In-hospital Complications** |  |  |  |  |  |  |  |  |  |  |
| No | 221 | 65 | — | — |  |  |  |  |  |  |
| Yes | 22 | 10 | 2.00 | 0.81, 4.87 | 0.126 |  |  |  |  |  |
| **Length of stay after operation** | 243 | 75 | 1.04 | 0.98, 1.12 | 0.205 | 243 | 75 | 1.08 | 1.00, 1.16 | 0.056 |
| OR = Odds Ratio, CI = Confidence Interval, BMI=Body Mass Index, FEV1%=Forced Expiratory Volume in the first second as a percentage of the predicted value, RUL= Right Upper Lobe | | | | | | | | | | |
|  | | | | | | | | | | |

## **Supplementary Table S3: Univariate and Multivariate Analysis of Factors Influencing Symptom Severity at Discharge – Cough**

| **Characteristic** | **Univariable** | | | | | **Multivariable** | | | | |
| --- | --- | --- | --- | --- | --- | --- | --- | --- | --- | --- |
|  | **N** | **Event N** | **OR** | **95% CI** | **p-value** | **N** | **Event N** | **OR** | **95% CI** | **p-value** |
| **Age, years** | 243 | 45 | 1.03 | 1.00, 1.07 | 0.047 | 243 | 45 | 1.03 | 0.99, 1.07 | 0.139 |
| **Gender** |  |  |  |  |  |  |  |  |  |  |
| Female | 129 | 27 | — | — |  | 129 | 27 | — | — |  |
| Male | 114 | 18 | 0.71 | 0.36, 1.36 | 0.305 | 114 | 18 | 0.31 | 0.11, 0.74 | 0.013 |
| **BMI, kg/m^2^** | 243 | 45 | 0.95 | 0.87, 1.04 | 0.289 |  |  |  |  |  |
| **Educational level** |  |  |  |  |  |  |  |  |  |  |
| Middle school or below/Unknown | 124 | 23 | — | — |  |  |  |  |  |  |
| Above middle school | 119 | 22 | 1.00 | 0.52, 1.91 | 0.990 |  |  |  |  |  |
| **Current smoking** |  |  |  |  |  |  |  |  |  |  |
| No | 200 | 32 | — | — |  | 200 | 32 | — | — |  |
| Yes | 43 | 13 | 2.28 | 1.05, 4.77 | 0.032 | 43 | 13 | 3.65 | 1.25, 11.09 | 0.019 |
| **Comorbidities** |  |  |  |  |  |  |  |  |  |  |
| No | 167 | 32 | — | — |  |  |  |  |  |  |
| Yes | 76 | 13 | 0.87 | 0.42, 1.74 | 0.702 |  |  |  |  |  |
| **FEV1%, %** | 237 | 44 | 1.00 | 0.98, 1.02 | 0.894 |  |  |  |  |  |
| **Extent of the procedure** |  |  |  |  |  |  |  |  |  |  |
| Sub-lobar | 135 | 22 | — | — |  | 135 | 22 | — | — |  |
| Lobectomy | 108 | 23 | 1.39 | 0.73, 2.67 | 0.320 | 108 | 23 | 0.84 | 0.38, 1.82 | 0.666 |
| **RUL involvement** |  |  |  |  |  |  |  |  |  |  |
| No | 171 | 16 | — | — |  | 171 | 16 | — | — |  |
| Yes | 72 | 29 | 6.53 | 3.29, 13.39 | <0.001 | 72 | 29 | 6.90 | 3.33, 14.89 | <0.001 |
| **Systematic dissection** |  |  |  |  |  |  |  |  |  |  |
| No | 51 | 10 | — | — |  |  |  |  |  |  |
| Yes | 192 | 35 | 0.91 | 0.43, 2.09 | 0.822 |  |  |  |  |  |
| **Operation time, minutes** | 243 | 45 | 1.00 | 1.00, 1.01 | 0.692 |  |  |  |  |  |
| **Tumor pathologic stage** |  |  |  |  |  |  |  |  |  |  |
| Benign or other tumor | 40 | 8 | — | — |  |  |  |  |  |  |
| Tis | 25 | 3 | 0.55 | 0.11, 2.12 | 0.407 |  |  |  |  |  |
| I | 151 | 28 | 0.91 | 0.39, 2.31 | 0.834 |  |  |  |  |  |
| II~III | 27 | 6 | 1.14 | 0.33, 3.76 | 0.826 |  |  |  |  |  |
| **In-hospital Complications** |  |  |  |  |  |  |  |  |  |  |
| No | 221 | 36 | — | — |  | 221 | 36 | — | — |  |
| Yes | 22 | 9 | 3.56 | 1.38, 8.89 | 0.007 | 22 | 9 | 2.41 | 0.74, 7.59 | 0.134 |
| **Length of stay after operation** | 243 | 45 | 1.08 | 1.00, 1.16 | 0.038 |  |  |  |  |  |
| OR = Odds Ratio, CI = Confidence Interval, BMI=Body Mass Index, FEV1%=Forced Expiratory Volume in the first second as a percentage of the predicted value, RUL= Right Upper Lobe | | | | | | | | | | |
|  | | | | | | | | | | |

## **Supplementary Table S4: Univariate and Multivariate Analysis of Factors Influencing Symptom Severity at Discharge – Shortness of Breath**

| **Characteristic** | **Univariable** | | | | | **Multivariable** | | | | |
| --- | --- | --- | --- | --- | --- | --- | --- | --- | --- | --- |
|  | **N** | **Event N** | **OR** | **95% CI** | **p-value** | **N** | **Event N** | **OR** | **95% CI** | **p-value** |
| **Age, years** | 243 | 39 | 1.04 | 1.00, 1.08 | 0.031 | 237 | 38 | 1.03 | 0.99, 1.07 | 0.105 |
| **Gender** |  |  |  |  |  |  |  |  |  |  |
| Female | 129 | 18 | — | — |  | 125 | 18 | — | — |  |
| Male | 114 | 21 | 1.39 | 0.70, 2.79 | 0.345 | 112 | 20 | 0.93 | 0.42, 1.99 | 0.846 |
| **BMI, kg/m^2^** | 243 | 39 | 0.99 | 0.90, 1.09 | 0.878 |  |  |  |  |  |
| **Educational level** |  |  |  |  |  |  |  |  |  |  |
| Middle school or below/Unknown | 124 | 24 | — | — |  |  |  |  |  |  |
| Above middle school | 119 | 15 | 0.60 | 0.29, 1.20 | 0.155 |  |  |  |  |  |
| **Current smoking** |  |  |  |  |  |  |  |  |  |  |
| No | 200 | 28 | — | — |  |  |  |  |  |  |
| Yes | 43 | 11 | 2.11 | 0.93, 4.58 | 0.065 |  |  |  |  |  |
| **Comorbidities** |  |  |  |  |  |  |  |  |  |  |
| No | 167 | 28 | — | — |  |  |  |  |  |  |
| Yes | 76 | 11 | 0.84 | 0.38, 1.75 | 0.652 |  |  |  |  |  |
| **FEV1%, %** | 237 | 38 | 0.98 | 0.96, 1.00 | 0.046 | 237 | 38 | 0.99 | 0.96, 1.01 | 0.205 |
| **Extent of the procedure** |  |  |  |  |  |  |  |  |  |  |
| Sub-lobar | 135 | 17 | — | — |  |  |  |  |  |  |
| Lobectomy | 108 | 22 | 1.78 | 0.89, 3.59 | 0.104 |  |  |  |  |  |
| **RUL involvement** |  |  |  |  |  |  |  |  |  |  |
| No | 171 | 23 | — | — |  |  |  |  |  |  |
| Yes | 72 | 16 | 1.84 | 0.89, 3.72 | 0.092 |  |  |  |  |  |
| **Systematic dissection** |  |  |  |  |  |  |  |  |  |  |
| No | 51 | 5 | — | — |  |  |  |  |  |  |
| Yes | 192 | 34 | 1.98 | 0.79, 6.03 | 0.178 |  |  |  |  |  |
| **Operation time, minutes** | 243 | 39 | 1.00 | 1.00, 1.01 | 0.439 |  |  |  |  |  |
| **Tumor pathologic stage** |  |  |  |  |  |  |  |  |  |  |
| Benign or other tumor | 40 | 5 | — | — |  |  |  |  |  |  |
| Tis | 25 | 4 | 1.33 | 0.30, 5.59 | 0.692 |  |  |  |  |  |
| I | 151 | 23 | 1.26 | 0.48, 3.95 | 0.665 |  |  |  |  |  |
| II~III | 27 | 7 | 2.45 | 0.69, 9.27 | 0.167 |  |  |  |  |  |
| **In-hospital Complications** |  |  |  |  |  |  |  |  |  |  |
| No | 221 | 30 | — | — |  |  |  |  |  |  |
| Yes | 22 | 9 | 4.41 | 1.69, 11.15 | 0.002 |  |  |  |  |  |
| **Length of stay after operation** | 243 | 39 | 1.13 | 1.05, 1.22 | 0.002 | 237 | 38 | 1.12 | 1.03, 1.21 | 0.007 |
| OR = Odds Ratio, CI = Confidence Interval, BMI=Body Mass Index, FEV1%=Forced Expiratory Volume in the first second as a percentage of the predicted value, RUL= Right Upper Lobe | | | | | | | | | | |
|  | | | | | | | | | | |

## **Supplementary Table S5: Univariate and Multivariate Analysis of Factors Influencing Symptom Severity at Discharge – Disturbed Sleep**

| **Characteristic** | **Univariable** | | | | | **Multivariable** | | | | |
| --- | --- | --- | --- | --- | --- | --- | --- | --- | --- | --- |
|  | **N** | **Event N** | **OR** | **95% CI** | **p-value** | **N** | **Event N** | **OR** | **95% CI** | **p-value** |
| **Age, years** | 243 | 71 | 1.01 | 0.98, 1.04 | 0.493 | 243 | 71 | 0.99 | 0.96, 1.02 | 0.627 |
| **Gender** |  |  |  |  |  |  |  |  |  |  |
| Female | 129 | 42 | — | — |  | 129 | 42 | — | — |  |
| Male | 114 | 29 | 0.71 | 0.40, 1.24 | 0.224 | 114 | 29 | 0.58 | 0.31, 1.07 | 0.081 |
| **BMI, kg/m^2^** | 243 | 71 | 1.03 | 0.95, 1.11 | 0.526 | 243 | 71 | 1.07 | 0.98, 1.16 | 0.135 |
| **Educational level** |  |  |  |  |  |  |  |  |  |  |
| Middle school or below/Unknown | 124 | 35 | — | — |  |  |  |  |  |  |
| Above middle school | 119 | 36 | 1.10 | 0.63, 1.92 | 0.728 |  |  |  |  |  |
| **Current smoking** |  |  |  |  |  |  |  |  |  |  |
| No | 200 | 56 | — | — |  |  |  |  |  |  |
| Yes | 43 | 15 | 1.38 | 0.68, 2.77 | 0.369 |  |  |  |  |  |
| **Comorbidities** |  |  |  |  |  |  |  |  |  |  |
| No | 167 | 50 | — | — |  |  |  |  |  |  |
| Yes | 76 | 21 | 0.89 | 0.49, 1.63 | 0.714 |  |  |  |  |  |
| **FEV1%, %** | 237 | 71 | 1.01 | 0.99, 1.02 | 0.446 |  |  |  |  |  |
| **Extent of the procedure** |  |  |  |  |  |  |  |  |  |  |
| Sub-lobar | 135 | 33 | — | — |  |  |  |  |  |  |
| Lobectomy | 108 | 38 | 1.68 | 0.96, 2.93 | 0.068 |  |  |  |  |  |
| **RUL involvement** |  |  |  |  |  |  |  |  |  |  |
| No | 171 | 41 | — | — |  | 171 | 41 | — | — |  |
| Yes | 72 | 30 | 2.26 | 1.26, 4.07 | 0.006 | 72 | 30 | 2.29 | 1.23, 4.28 | 0.009 |
| **Systematic dissection** |  |  |  |  |  |  |  |  |  |  |
| No | 51 | 13 | — | — |  |  |  |  |  |  |
| Yes | 192 | 58 | 1.27 | 0.63, 2.55 | 0.511 |  |  |  |  |  |
| **Operation time, minutes** | 243 | 71 | 1.00 | 1.00, 1.01 | 0.431 |  |  |  |  |  |
| **Tumor pathologic stage** |  |  |  |  |  |  |  |  |  |  |
| Benign or other tumor | 40 | 8 | — | — |  | 40 | 8 | — | — |  |
| Tis | 25 | 3 | 0.55 | 0.13, 2.29 | 0.407 | 25 | 3 | 0.63 | 0.14, 2.78 | 0.543 |
| I | 151 | 47 | 1.81 | 0.77, 4.22 | 0.171 | 151 | 47 | 1.74 | 0.71, 4.28 | 0.229 |
| II~III | 27 | 13 | 3.71 | 1.26, 10.96 | 0.017 | 27 | 13 | 4.51 | 1.42, 14.35 | 0.011 |
| **In-hospital Complications** |  |  |  |  |  |  |  |  |  |  |
| No | 221 | 59 | — | — |  | 221 | 59 | — | — |  |
| Yes | 22 | 12 | 3.29 | 1.35, 8.03 | 0.009 | 22 | 12 | 3.24 | 1.23, 8.56 | 0.017 |
| **Length of stay after operation** | 243 | 71 | 1.09 | 1.02, 1.17 | 0.015 |  |  |  |  |  |
| OR = Odds Ratio, CI = Confidence Interval, BMI=Body Mass Index, FEV1%=Forced Expiratory Volume in the first second as a percentage of the predicted value, RUL= Right Upper Lobe | | | | | | | | | | |
|  | | | | | | | | | | |

## **Supplementary Table S6: Univariate and Multivariate Analysis of Factors Influencing Symptom Severity at Discharge – Fatigue**

| **Characteristic** | **Univariable** | | | | | **Multivariable** | | | | |
| --- | --- | --- | --- | --- | --- | --- | --- | --- | --- | --- |
|  | **N** | **Event N** | **OR** | **95% CI** | **p-value** | **N** | **Event N** | **OR** | **95% CI** | **p-value** |
| **v** | 243 | 63 | 1.01 | 0.98, 1.04 | 0.577 | 243 | 63 | 0.99 | 0.96, 1.02 | 0.662 |
| **Gender** |  |  |  |  |  |  |  |  |  |  |
| Female | 129 | 35 | — | — |  | 129 | 35 | — | — |  |
| Male | 114 | 28 | 0.87 | 0.49, 1.55 | 0.648 | 114 | 28 | 0.73 | 0.39, 1.34 | 0.318 |
| **BMI, kg/m^2^** | 243 | 63 | 0.99 | 0.92, 1.08 | 0.860 |  |  |  |  |  |
| **Educational level** |  |  |  |  |  |  |  |  |  |  |
| Middle school or below/Unknown | 124 | 35 | — | — |  |  |  |  |  |  |
| Above middle school | 119 | 28 | 0.78 | 0.44, 1.39 | 0.404 |  |  |  |  |  |
| **Current smoking** |  |  |  |  |  |  |  |  |  |  |
| No | 200 | 50 | — | — |  |  |  |  |  |  |
| Yes | 43 | 13 | 1.30 | 0.61, 2.64 | 0.478 |  |  |  |  |  |
| **Comorbidities** |  |  |  |  |  |  |  |  |  |  |
| No | 167 | 41 | — | — |  |  |  |  |  |  |
| Yes | 76 | 22 | 1.25 | 0.67, 2.29 | 0.469 |  |  |  |  |  |
| **FEV1%^1^, %** | 237 | 61 | 0.99 | 0.98, 1.01 | 0.427 |  |  |  |  |  |
| **Extent of the procedure** |  |  |  |  |  |  |  |  |  |  |
| Sub-lobar | 135 | 27 | — | — |  | 135 | 27 | — | — |  |
| Lobectomy | 108 | 36 | 2.00 | 1.12, 3.60 | 0.019 | 108 | 36 | 1.93 | 1.00, 3.79 | 0.052 |
| **RUL involvement** |  |  |  |  |  |  |  |  |  |  |
| No | 171 | 41 | — | — |  |  |  |  |  |  |
| Yes | 72 | 22 | 1.40 | 0.75, 2.56 | 0.286 |  |  |  |  |  |
| **Systematic dissection** |  |  |  |  |  |  |  |  |  |  |
| No | 51 | 15 | — | — |  | 51 | 15 | — | — |  |
| Yes | 192 | 48 | 0.80 | 0.41, 1.62 | 0.523 | 192 | 48 | 0.55 | 0.26, 1.18 | 0.120 |
| **Operation time, minutes** | 243 | 63 | 1.00 | 1.00, 1.01 | 0.160 |  |  |  |  |  |
| **Tumor pathologic stage** |  |  |  |  |  |  |  |  |  |  |
| Benign or other tumor | 40 | 9 | — | — |  |  |  |  |  |  |
| Tis | 25 | 3 | 0.47 | 0.10, 1.78 | 0.296 |  |  |  |  |  |
| I | 151 | 40 | 1.24 | 0.56, 2.97 | 0.608 |  |  |  |  |  |
| II~III | 27 | 11 | 2.37 | 0.82, 7.06 | 0.114 |  |  |  |  |  |
| **In-hospital Complications** |  |  |  |  |  |  |  |  |  |  |
| No | 221 | 50 | — | — |  | 221 | 50 | — | — |  |
| Yes | 22 | 13 | 4.94 | 2.02, 12.62 | <0.001 | 22 | 13 | 4.80 | 1.84, 13.06 | 0.002 |
| **Length of stay after operation** | 243 | 63 | 1.10 | 1.02, 1.18 | 0.011 |  |  |  |  |  |
| OR = Odds Ratio, CI = Confidence Interval, BMI=Body Mass Index, FEV1%=Forced Expiratory Volume in the first second as a percentage of the predicted value, RUL= Right Upper Lobe | | | | | | | | | | |
|  | | | | | | | | | | |

## **Supplementary Table S7: Univariate and Multivariate Analysis of Factors Influencing Symptom Severity at Discharge – Drowsiness**

| **Characteristic** | **Univariable** | | | | | **Multivariable** | | | | |
| --- | --- | --- | --- | --- | --- | --- | --- | --- | --- | --- |
|  | **N** | **Event N** | **OR** | **95% CI** | **p-value** | **N** | **Event N** | **OR** | **95% CI** | **p-value** |
| **Age, years** | 243 | 48 | 1.01 | 0.98, 1.05 | 0.369 | 243 | 48 | 1.00 | 0.97, 1.03 | 0.889 |
| **Gender** |  |  |  |  |  |  |  |  |  |  |
| Female | 129 | 24 | — | — |  | 129 | 24 | — | — |  |
| Male | 114 | 24 | 1.17 | 0.62, 2.20 | 0.633 | 114 | 24 | 0.96 | 0.49, 1.88 | 0.916 |
| **BMI, kg/m^2^** | 243 | 48 | 1.03 | 0.94, 1.13 | 0.528 |  |  |  |  |  |
| **Educational level** |  |  |  |  |  |  |  |  |  |  |
| Middle school or below/Unknown | 124 | 27 | — | — |  |  |  |  |  |  |
| Above middle school | 119 | 21 | 0.77 | 0.40, 1.45 | 0.420 |  |  |  |  |  |
| **Current smoking** |  |  |  |  |  |  |  |  |  |  |
| No | 200 | 35 | — | — |  |  |  |  |  |  |
| Yes | 43 | 13 | 2.04 | 0.95, 4.25 | 0.061 |  |  |  |  |  |
| **Comorbidities** |  |  |  |  |  |  |  |  |  |  |
| No | 167 | 29 | — | — |  |  |  |  |  |  |
| Yes | 76 | 19 | 1.59 | 0.81, 3.04 | 0.168 |  |  |  |  |  |
| **FEV1%, %** | 237 | 48 | 0.99 | 0.97, 1.01 | 0.329 |  |  |  |  |  |
| **Extent of the procedure** |  |  |  |  |  |  |  |  |  |  |
| Sub-lobar | 135 | 23 | — | — |  |  |  |  |  |  |
| Lobectomy | 108 | 25 | 1.47 | 0.78, 2.78 | 0.236 |  |  |  |  |  |
| **RUL involvement** |  |  |  |  |  |  |  |  |  |  |
| No | 171 | 29 | — | — |  |  |  |  |  |  |
| Yes | 72 | 19 | 1.76 | 0.90, 3.38 | 0.094 |  |  |  |  |  |
| **Systematic dissection** |  |  |  |  |  |  |  |  |  |  |
| No | 51 | 8 | — | — |  |  |  |  |  |  |
| Yes | 192 | 40 | 1.41 | 0.64, 3.46 | 0.414 |  |  |  |  |  |
| **Operation time, minutes** | 243 | 48 | 1.00 | 1.00, 1.01 | 0.368 |  |  |  |  |  |
| **Tumor pathologic stage** |  |  |  |  |  |  |  |  |  |  |
| Benign or other tumor | 40 | 7 | — | — |  |  |  |  |  |  |
| Tis | 25 | 1 | 0.20 | 0.01, 1.21 | 0.140 |  |  |  |  |  |
| I | 151 | 35 | 1.42 | 0.61, 3.75 | 0.442 |  |  |  |  |  |
| II~III | 27 | 5 | 1.07 | 0.29, 3.79 | 0.915 |  |  |  |  |  |
| **In-hospital Complications** |  |  |  |  |  |  |  |  |  |  |
| No | 221 | 36 | — | — |  | 221 | 36 | — | — |  |
| Yes | 22 | 12 | 6.17 | 2.48, 15.68 | <0.001 | 22 | 12 | 6.13 | 2.38, 16.18 | <0.001 |
| **Length of stay after operation** | 243 | 48 | 1.08 | 1.00, 1.16 | 0.037 |  |  |  |  |  |
| OR = Odds Ratio, CI = Confidence Interval, BMI=Body Mass Index, FEV1%=Forced Expiratory Volume in the first second as a percentage of the predicted value, RUL= Right Upper Lobe | | | | | | | | | | |
|  | | | | | | | | | | |

## **Supplementary Table S8: Univariate and Multivariate Analysis of Factors Influencing Symptom Severity at Discharge – Sadness**

| **Characteristic** | **Univariable** | | | | | **Multivariable** | | | | |
| --- | --- | --- | --- | --- | --- | --- | --- | --- | --- | --- |
|  | **N** | **Event N** | **OR** | **95% CI** | **p-value** | **N** | **Event N** | **OR** | **95% CI** | **p-value** |
| **Age, years** | 243 | 33 | 1.01 | 0.98, 1.05 | 0.551 | 243 | 33 | 0.99 | 0.95, 1.03 | 0.529 |
| **Gender** |  |  |  |  |  |  |  |  |  |  |
| Female | 129 | 20 | — | — |  | 129 | 20 | — | — |  |
| Male | 114 | 13 | 0.70 | 0.32, 1.47 | 0.353 | 114 | 13 | 0.39 | 0.15, 0.92 | 0.039 |
| **BMI, kg/m^2^** | 243 | 33 | 1.04 | 0.94, 1.16 | 0.463 | 243 | 33 | 1.10 | 0.98, 1.24 | 0.110 |
| **Educational level** |  |  |  |  |  |  |  |  |  |  |
| Middle school or below/Unknown | 124 | 20 | — | — |  |  |  |  |  |  |
| Above middle school | 119 | 13 | 0.64 | 0.30, 1.34 | 0.239 |  |  |  |  |  |
| **Current smoking** |  |  |  |  |  |  |  |  |  |  |
| No | 200 | 25 | — | — |  |  |  |  |  |  |
| Yes | 43 | 8 | 1.60 | 0.63, 3.71 | 0.292 |  |  |  |  |  |
| **Comorbidities** |  |  |  |  |  |  |  |  |  |  |
| No | 167 | 24 | — | — |  |  |  |  |  |  |
| Yes | 76 | 9 | 0.80 | 0.34, 1.76 | 0.594 |  |  |  |  |  |
| **FEV1%, %** | 237 | 33 | 0.99 | 0.97, 1.02 | 0.557 |  |  |  |  |  |
| **Extent of the procedure** |  |  |  |  |  |  |  |  |  |  |
| Sub-lobar | 135 | 15 | — | — |  |  |  |  |  |  |
| Lobectomy | 108 | 18 | 1.60 | 0.77, 3.39 | 0.212 |  |  |  |  |  |
| **RUL involvement** |  |  |  |  |  |  |  |  |  |  |
| No | 171 | 21 | — | — |  |  |  |  |  |  |
| Yes | 72 | 12 | 1.43 | 0.65, 3.05 | 0.364 |  |  |  |  |  |
| **Systematic dissection** |  |  |  |  |  |  |  |  |  |  |
| No | 51 | 4 | — | — |  |  |  |  |  |  |
| Yes | 192 | 29 | 2.09 | 0.77, 7.31 | 0.187 |  |  |  |  |  |
| **Operation time, minutes** | 243 | 33 | 1.00 | 1.00, 1.01 | 0.533 |  |  |  |  |  |
| **Tumor pathologic stage** |  |  |  |  |  |  |  |  |  |  |
| Benign or other tumor | 40 | 2 | — | — |  |  |  |  |  |  |
| Tis | 25 | 2 | 1.65 | 0.19, 14.55 | 0.627 |  |  |  |  |  |
| I | 151 | 25 | 3.77 | 1.06, 24.10 | 0.080 |  |  |  |  |  |
| II~III | 27 | 4 | 3.30 | 0.60, 25.21 | 0.187 |  |  |  |  |  |
| **In-hospital Complications** |  |  |  |  |  |  |  |  |  |  |
| No | 221 | 22 | — | — |  | 221 | 22 | — | — |  |
| Yes | 22 | 11 | 9.05 | 3.50, 23.60 | <0.001 | 22 | 11 | 6.86 | 1.92, 25.29 | 0.003 |
| **Length of stay after operation** | 243 | 33 | 1.16 | 1.08, 1.27 | <0.001 | 243 | 33 | 1.11 | 1.00, 1.24 | 0.057 |
| OR = Odds Ratio, CI = Confidence Interval, BMI=Body Mass Index, FEV1%=Forced Expiratory Volume in the first second as a percentage of the predicted value, RUL= Right Upper Lobe | | | | | | | | | | |
|  | | | | | | | | | | |

## **Supplementary Table S9: Univariate and Multivariate Analysis of Factors Influencing Symptom Severity at Discharge – Walking Ability**

| **Characteristic** | **Univariable** | | | | | **Multivariable** | | | | |
| --- | --- | --- | --- | --- | --- | --- | --- | --- | --- | --- |
|  | **N** | **Event N** | **OR** | **95% CI** | **p-value** | **N** | **Event N** | **OR** | **95% CI** | **p-value** |
| **Age, years** | 243 | 42 | 1.06 | 1.03, 1.10 | <0.001 | 237 | 42 | 1.07 | 1.03, 1.12 | 0.002 |
| **Gender** |  |  |  |  |  |  |  |  |  |  |
| Female | 129 | 21 | — | — |  | 125 | 21 | — | — |  |
| Male | 114 | 21 | 1.16 | 0.59, 2.27 | 0.660 | 112 | 21 | 0.59 | 0.25, 1.32 | 0.208 |
| **BMI, kg/m^2^** | 243 | 42 | 0.96 | 0.88, 1.05 | 0.388 |  |  |  |  |  |
| **Educational level** |  |  |  |  |  |  |  |  |  |  |
| Middle school or below/Unknown | 124 | 21 | — | — |  | 120 | 21 | — | — |  |
| Above middle school | 119 | 21 | 1.05 | 0.54, 2.05 | 0.883 | 117 | 21 | 3.06 | 1.28, 7.72 | 0.014 |
| **Current smoking** |  |  |  |  |  |  |  |  |  |  |
| No | 200 | 31 | — | — |  |  |  |  |  |  |
| Yes | 43 | 11 | 1.87 | 0.83, 4.03 | 0.117 |  |  |  |  |  |
| **Comorbidities** |  |  |  |  |  |  |  |  |  |  |
| No | 167 | 26 | — | — |  |  |  |  |  |  |
| Yes | 76 | 16 | 1.45 | 0.71, 2.87 | 0.296 |  |  |  |  |  |
| **FEV1%, %** | 237 | 42 | 0.97 | 0.95, 0.99 | 0.004 | 237 | 42 | 0.98 | 0.95, 1.00 | 0.047 |
| **Extent of the procedure** |  |  |  |  |  |  |  |  |  |  |
| Sub-lobar | 135 | 19 | — | — |  |  |  |  |  |  |
| Lobectomy | 108 | 23 | 1.65 | 0.85, 3.25 | 0.141 |  |  |  |  |  |
| **RUL involvement** |  |  |  |  |  |  |  |  |  |  |
| No | 171 | 25 | — | — |  |  |  |  |  |  |
| Yes | 72 | 17 | 1.81 | 0.89, 3.58 | 0.093 |  |  |  |  |  |
| **Systematic dissection** |  |  |  |  |  |  |  |  |  |  |
| No | 51 | 8 | — | — |  |  |  |  |  |  |
| Yes | 192 | 34 | 1.16 | 0.52, 2.85 | 0.734 |  |  |  |  |  |
| **Operation time, minutes** | 243 | 42 | 1.00 | 1.00, 1.01 | 0.220 |  |  |  |  |  |
| **Tumor pathologic stage** |  |  |  |  |  |  |  |  |  |  |
| Benign or other tumor | 40 | 8 | — | — |  |  |  |  |  |  |
| Tis | 25 | 4 | 0.76 | 0.18, 2.75 | 0.686 |  |  |  |  |  |
| I | 151 | 23 | 0.72 | 0.30, 1.85 | 0.469 |  |  |  |  |  |
| II~III | 27 | 7 | 1.40 | 0.43, 4.50 | 0.569 |  |  |  |  |  |
| **In-hospital Complications** |  |  |  |  |  |  |  |  |  |  |
| No | 221 | 27 | — | — |  | 215 | 27 | — | — |  |
| Yes | 22 | 15 | 15.40 | 5.95, 43.60 | <0.001 | 22 | 15 | 15.19 | 5.21, 49.18 | <0.001 |
| **Length of stay after operation** | 243 | 42 | 1.17 | 1.08, 1.27 | <0.001 |  |  |  |  |  |
| OR = Odds Ratio, CI = Confidence Interval, BMI=Body Mass Index, FEV1%=Forced Expiratory Volume in the first second as a percentage of the predicted value, RUL= Right Upper Lobe | | | | | | | | | | |
|  | | | | | | | | | | |

## **Supplementary Table S10: Univariate and Multivariate Analysis of Factors Influencing Symptom Severity at Discharge – General Activity**

| **Characteristic** | **Univariable** | | | | | **Multivariable** | | | | |  |
| --- | --- | --- | --- | --- | --- | --- | --- | --- | --- | --- | --- |
|  | **N** | **Event N** | **OR** | **95% CI** | **p-value** | **N** | **Event N** | **OR** | **95% CI** | **p-value** |  |
| **Age, years** | 243 | 43 | 1.05 | 1.02, 1.09 | 0.004 | 243 | 43 | 1.04 | 1.00, 1.08 | 0.035 |  |
| **Gender** |  |  |  |  |  |  |  |  |  |  |  |
| Female | 129 | 25 | — | — |  | 129 | 25 | — | — |  |  |
| Male | 114 | 18 | 0.78 | 0.40, 1.51 | 0.465 | 114 | 18 | 0.50 | 0.22, 1.06 | 0.077 |  |
| **BMI, kg/m^2^** | 243 | 43 | 0.99 | 0.90, 1.08 | 0.799 |  |  |  |  |  |  |
| **Educational level** |  |  |  |  |  |  |  |  |  |  |  |
| Middle school or below/Unknown | 124 | 25 | — | — |  |  |  |  |  |  |  |
| Above middle school | 119 | 18 | 0.71 | 0.36, 1.37 | 0.305 |  |  |  |  |  |  |
| **Current smoking** |  |  |  |  |  |  |  |  |  |  |  |
| No | 200 | 32 | — | — |  |  |  |  |  |  |  |
| Yes | 43 | 11 | 1.80 | 0.80, 3.87 | 0.139 |  |  |  |  |  |  |
| **Comorbidities** |  |  |  |  |  |  |  |  |  |  |  |
| No | 167 | 26 | — | — |  |  |  |  |  |  |  |
| Yes | 76 | 17 | 1.56 | 0.78, 3.08 | 0.200 |  |  |  |  |  |  |
| **FEV1%, %** | 237 | 43 | 0.98 | 0.96, 1.00 | 0.073 |  |  |  |  |  |  |
| **Extent of the procedure** |  |  |  |  |  |  |  |  |  |  |  |
| Sub-lobar | 135 | 19 | — | — |  |  |  |  |  |  |  |
| Lobectomy | 108 | 24 | 1.74 | 0.90, 3.42 | 0.101 |  |  |  |  |  |  |
| **RUL involvement** |  |  |  |  |  |  |  |  |  |  |  |
| No | 171 | 24 | — | — |  | 171 | 24 | — | — |  |  |
| Yes | 72 | 19 | 2.20 | 1.11, 4.33 | 0.023 | 72 | 19 | 1.83 | 0.85, 3.86 | 0.115 |  |
| **Systematic dissection** |  |  |  |  |  |  |  |  |  |  |  |
| No | 51 | 7 | — | — |  |  |  |  |  |  |  |
| Yes | 192 | 36 | 1.45 | 0.64, 3.75 | 0.405 |  |  |  |  |  |  |
| **Operation time, minutes** | 243 | 43 | 1.00 | 1.00, 1.01 | 0.106 |  |  |  |  |  |  |
| **Tumor pathologic stage** |  |  |  |  |  |  |  |  |  |  |  |
| Benign or other tumor | 40 | 7 | — | — |  |  |  |  |  |  |  |
| Tis | 25 | 2 | 0.41 | 0.06, 1.88 | 0.292 |  |  |  |  |  |  |
| I | 151 | 27 | 1.03 | 0.43, 2.74 | 0.955 |  |  |  |  |  |  |
| II~III | 27 | 7 | 1.65 | 0.50, 5.51 | 0.408 |  |  |  |  |  |  |
| **In-hospital Complications** |  |  |  |  |  |  |  |  |  |  |  |
| No | 221 | 30 | — | — |  | 221 | 30 | — | — |  |  |
| Yes | 22 | 13 | 9.20 | 3.66, 24.13 | <0.001 | 22 | 13 | 5.58 | 1.65, 19.75 | 0.006 |  |
| **Length of stay after operation** | 243 | 43 | 1.15 | 1.07, 1.25 | <0.001 | 243 | 43 | 1.06 | 0.95, 1.18 | 0.273 |  |
| OR = Odds Ratio, CI = Confidence Interval, BMI=Body Mass Index, FEV1%=Forced Expiratory Volume in the first second as a percentage of the predicted value, RUL= Right Upper Lobe | | | | | | | | | | | |
|  | | | | | | | | | | | |
